# Pregestational Diabetes and Duration of Active Labour Compared With Non‐Diabetic Women: A Population‐Based Cohort Study

**DOI:** 10.1111/1471-0528.18276

**Published:** 2025-07-07

**Authors:** Sofia Nevander, Sara Carlhäll, Karin Källén, Caroline Lilliecreutz, Marie Blomberg

**Affiliations:** ^1^ Department of Obstetrics and Gynecology and Department of Biomedical and Clinical Sciences Linköping University Linköping Sweden; ^2^ Institution of Clinical Sciences, Lund. Department of Obstetrics and Gynecology Lund University Lund Sweden

**Keywords:** caesarean section, diabetes, indication for caesarean section, labour, pregnancy

## Abstract

**Objective:**

To evaluate the impact of pregestational diabetes on duration of active labour (DAL) in induced and spontaneous labour and to compare caesarean section (CS) rates and indications between women with and without diabetes.

**Design:**

A population‐based cohort study.

**Setting:**

Sweden.

**Population:**

243 537 nulliparous women, registered in the Swedish Pregnancy Register, who delivered a singleton fetus at ≥ 34^+0^ gestational weeks + days between 2014 and 2020. Women with gestational diabetes mellitus were excluded.

**Methods:**

DAL was compared between women with pregestational diabetes and those without diabetes using Kaplan‐Meier survival analysis and Cox regression analysis.

**Main Outcome Measures:**

DAL. Rates and indications for CS.

**Results:**

Women with pregestational diabetes had longer active labour and a reduced chance of vaginal delivery at a given time‐point compared to women without diabetes, adjusted hazard ratio 0.65 (95% CI: 0.60–0.70, *p* < 0.001). Among those with spontaneous labour, median DAL in diabetic vs. non‐diabetic women was 9.60 h versus 8.75 h, difference 0.85 h (95% CI 0.20–1.50), *p* < 0.001. Corresponding numbers for induced labours were 8.92 h versus 7.20 h, difference 1.72 h (95% CI 0.94–2.49), *p* < 0.001.

Elective and emergency CS rates were higher in women with pregestational diabetes than non‐diabetic women (7.4% and 29.4% vs. 2.6% and 7.1% respectively), with suspected macrosomia (50.4%) and fetal distress (31.9%) being the most common indications for CS among women with pregestational diabetes.

**Conclusions:**

The prolonged labour duration in women with pregestational diabetes highlights the importance of the labour ward staff's support and patience in managing diabetic parturients, potentially allowing more time before diagnosing labour dystocia. Extended labour duration may also influence women's birth experience.

## 1 Introduction

Women with pregestational diabetes have increased rates of adverse obstetric and perinatal outcomes, including congenital malformations, preeclampsia, stillbirth, fetal macrosomia, and caesarean delivery [1, 2, 3, 4] compared to women without diabetes.

The risk of emergency caesarean section (CS) is 3–4 times higher compared to women without diabetes [5, 6] and despite therapeutic initiatives and technological advancements, the overall CS rate in women with pregestational diabetes remains persistently above 60% [4, 6, 7, 8].

A common indication for elective CS in women with pregestational diabetes is macrosomia [9]. While both fetal distress and macrosomia contribute to the increased emergency CS rate in these women, previous studies [4, 5] also indicate that labour arrest accounts for a substantial proportion of emergency CS among women with type 1 diabetes, with reported rates of 47.9% [4] and 51.6% [5] respectively.

In vitro studies of the myometrium in humans [10] and mice [11] comparing individuals with pregestational diabetes or gestational diabetes mellitus (GDM) to those without diabetes show that diabetes can lead to alterations in the myometrium, indicating impaired uterine contractility, which may partly explain [10] the increased emergency CS rate in women with diabetes.

Studies examining labour duration in women with pregestational diabetes are limited and have included mixed cohorts of women with pregestational diabetes and GDM [12, 13], primarily focusing on vaginal deliveries. Previous research suggests that the duration of active labour is comparable between women with diabetes (pregestational diabetes or GDM) and non‐diabetic controls [12, 13]. In contrast, one study reports that GDM alone is associated with a longer duration of active labour (DAL) [14]. Given that pregestational diabetes represents a higher‐risk profile than GDM during both pregnancy and labour [3], it is plausible that the duration of labour differs between these groups.

To clarify these inconsistencies, it is essential to specifically investigate active labour duration in a well‐defined cohort of women with pregestational diabetes and to include emergency CS in order to explore whether prolonged labour contributes to the elevated CS risk in this population.

We hypothesised that pregestational diabetes is associated with longer duration of active labour, in addition to other known factors influencing labour progression.

Therefore, the aims of this study were to evaluate the impact of pregestational diabetes on DAL in nulliparous women in induced and spontaneous onset of labour and to compare CS indications and rates, ‐both elective and emergency, ‐with those of women without diabetes.

## 2 Material and Methods

### 2.1 Population

Nulliparous women who delivered a singleton fetus with cephalic presentation between 1 January 2014 and 30 May 2020, ≥ 34^+0^ (completed gestational weeks + additional days), and had their data available in the Swedish Pregnancy Register (SPR) were included in the study (*N* = 243 537).

The SPR is a national quality register established in 2013. The SPR collects data on pregnancy and childbirth, from the first visit at the antenatal care clinic to the follow‐up visit 6–12 weeks postpartum. It contains data on maternal characteristics, pregnancy complications, and labour and birth outcomes. In 2023, 98.9% of all deliveries in Sweden were registered in the SPR compared to a coverage rate of 85% in 2014. The majority of variables included in the register are continuously transferred electronically from the medical antenatal and labour records, while some variables are manually entered by midwives at the antenatal care clinics [15].

### 2.2 Exclusions

Women with gestational diabetes were excluded. To define the study population for the analysis of DAL, we excluded all women delivered by elective CS. The dataset was thereby restricted to nulliparae women who underwent a trial of labour (TOL), including women who delivered by emergency CS. Subsequently, we excluded women with missing or faulty data regarding the onset of active labour. This resulted in a final study population comprising nulliparous women with valid information on the start of active labour who attempted a TOL (Figure S1).

### 2.3 Covariate Definitions

Pregestational diabetes included women with all types of pregestational diabetes, including type 1 diabetes, type 2 diabetes, and other more uncommon types of pregestational diabetes.

Gestational weight gain (GWG) was defined as the maternal weight increase from the initial baseline visit in early pregnancy to the measured weight upon admission to the delivery unit. The study population was sub‐classified into below, within, or above recommended GWG according to the American Institute of Medicines guidelines, in relation to pregestational body mass index (BMI) [16]. Gestational age at delivery was calculated using the estimated date of delivery by early second‐trimester ultrasound. When an ultrasound estimation was missing, the first day of the last menstrual period was used. Small for gestational age (SGA) was defined as birth weight below two standard deviations relative to gestational week and sex using Swedish growth charts. Large for gestational age (LGA) was defined as birth weight above two standard deviations relative to gestational week and sex using the Swedish growth charts [17].

### 2.4 Labour and Perinatal Variables

The birth variables assessed included type of labour onset (spontaneous/induced), time of start of active phase of labour, time of delivery, epidural anaesthesia, oxytocin administration, fetal presentation, instrumental delivery, mode of delivery, indications for both elective and emergency CS, fetal birthweight, small for gestational age (SGA), and large for gestational age (LGA). A definition of how all the different variables were extracted is presented in Table S1.

### 2.5 Outcomes

Outcomes studied were DAL and rates and indications for elective and emergency CS. DAL was defined as the time from the start of active labour until the time of delivery. From Jan 2014 to March 2015, the onset of active labour was defined as cervical dilatation of three centimetres or more in women with painful regular uterine contractions, in accordance with the previous Swedish national definition. During the remainder of the study period, the onset of active labour was defined according to the present Swedish national definition. This definition states that at least two out of three of the following criteria must be fulfilled: spontaneous rupture of the membranes, regular painful contractions (2–3/10 min), and a cervical dilatation of four centimetres or cervix effaced and dilated more than 1 cm. Additionally, labour should progress within the subsequent 2 h [18].

DAL was compared between women with pregestational diabetes and women without any type of diabetes.

Data on rates and indications for elective and emergency CS were analysed separately.

To address cases where women had multiple indications for CS, two hierarchies of indications, one for elective CS and one for emergency CS, were developed in collaboration with experienced obstetricians at Linköping University Hospital. These hierarchies aimed to prioritise the most clinically relevant indication for CS for each woman with pregestational diabetes, ensuring that each CS was counted only once. This approach provided a clearer distribution of CS indications. The clinical relevance of indications was specifically assessed for women with pregestational diabetes. The number of CS in each indication group includes CS with that specific indication but without the number of CS due to the indications listed above in the hierarchy. For example, suspected macrosomia, considered the most important indication for CS, included all CS performed for this reason, regardless of other indications. The second most important indication for CS, preeclampsia, included CS for preeclampsia only if macrosomia was not diagnosed, and so on down the hierarchy. This method ensured that each CS was attributed to the most relevant indication based on the predefined order of importance. The CS indication hierarchies for elective and emergency CS are presented in Table S2.

### 2.6 Statistical Analyses

#### 2.6.1 Descriptive Data

Categorical data are presented as numbers and percentages, and differences between groups were evaluated by Chi‐square analyses. Continuous data in descriptive tables were presented as means and SD, and the differences between groups were evaluated with Mann–Whitney *U*‐test.

#### 2.6.2 Analyses

Difference of CS rates and indications between groups were expressed as Risk Ratios and were obtained using modified Poisson‐regression analyses.

Kaplan–Meier survival analyses were performed, and graphs were produced to illustrate the association between pregestational diabetes and DAL, taking censoring due to emergency CS into account when applicable. Women with spontaneous labour onset and women with induced labour were analysed separately in the Kaplan–Meier analyses, and differences between groups were evaluated using Log‐Rank tests.

Adjusted hazard ratios (aHR) for labour at a certain time‐point were retrieved using Cox regression analyses, considering the time from start of active labour until the time of labour, or time of emergency CS, respectively. Confounding factors that were adjusted for included early pregnancy maternal BMI (classes, one class consisted of missing BMI information), smoking (yes/no/missing), maternal age (in years, continuous), gestational week at delivery (in weeks, continuous) and induction of labour (yes/no). Birth weight was considered a mediator and was adjusted for in a separate analysis (in grams, continuous). Confounding factors and mediators were assessed using a directed acyclic graph (Figure S2).

#### 2.6.3 Missing Data

Missing information on maternal smoking was replaced by the overall mean. BMI was entered into the model as a class variable, including all women with missing BMI into one class. Drop‐out analyses in TOL deliveries were conducted to compare women with and without available data on the start of active labour. Chi‐square analyses were used to calculate the overall heterogeneity within each domain.

The statistical analyses were performed using IBM SPSS version 28 (IMB inc., Armok, NY). A *p*‐value < 0.05 was considered statistically significant.

This study was approved by the Regional Ethical Review Board in Linköping, Sweden (2018/464–31), date of approval 2018‐11‐06. Data were de‐identified, and no direct patient participation was involved; therefore, informed consent was waived.

## 3 Results

A total of 167 650 nulliparous women were included in the final study population for analyses of DAL, and 832 (0.5%) of these women had pregestational diabetes. A total of 6262 women had an elective CS, and 115 (1.8%) of these women had pregestational diabetes. After excluding women with elective CS and women with GDM, 27.8% of the remaining women had missing data on the time of the start of active labour and were also excluded from the final study population for analysis of DAL. The cohort collection process is summarised and visualised in Figure S1. Drop‐out analyses and characteristics of the women who had missing data on the time of the start of active labour did not show any clinically relevant demographic difference (Table S3).

Maternal, labour, and neonatal characteristics for women with TOL and those undergoing elective CS are presented in Table 1. Among women with TOL, women with diabetes were more likely to be overweight or obese, and to gain more than the recommended weight during pregnancy, compared to women with no diabetes. Women with diabetes had shorter gestations, more often induction of labour, and had a higher risk to give birth to an LGA infant (26.2%, compared to 2.0% in the group of women with no diabetes).

**TABLE 1:** Maternal, labour and neonatal characteristics for women with trial of labour (with available data on start of active labour) and women undergoing elective caesarean section.

	Elective caesarean section			Trial of labour		
	Pre‐gestational diabetes *N* = 115	No diabetes *N* = 6147	*p* ^a^	Pre‐gestational diabetes *N* = 832	No diabetes *N* = 166 818	*p* ^a^
	*n* (%)	*n* (%)		*n* (%)	*n* (%)	
Age (years) mean, [SD]	31.7 [5.6]	32.8 [5.7]	0.043^b^	29.4 [4.7]	28.9 [4.8]	0.027^b^
Country of birth			0.015			0.015
Nordic countries	86 (74.8)	4231 (68.8)		593 (71.3)	118 821 (71.2)	
Other EU and USA	3 (2.6)	388 (6.3)		35 (4.2)	10 801 (6.5)	
Remaining/other countries	26 (22.6)	1528 (24.9)		204 (24.5)	37 196 (22.3)	
BMI (kg/m^2^) in early pregnancy			< 0.001			< 0.001
< 18.5	1 (0.9)	197 (3.2)		9 (1.1)	4825 (2.9)	
18.5–24.9	38 (33.0)	3215 (52.3)		355 (42.7)	95 005 (57.0)	
25–29.9	33 (28.7)	1217 (19.8)		229 (27.5)	34 401 (20.6)	
30–34.9	20 (17.4)	462 (7.5)		100 (12.0)	10 840 (6.5)	
≥ 35	7 (6.1)	164 (2.7)		49 (5.9)	4038 (2.4)	
BMI unknown	16 (13.9)	892 (14.5)		90 (10.8)	17 709 (10.6)	
Gestational weight gain (gwg)^c^						
Below recommended gwg	21 (18.3)	1560 (25.4)	0.049	159 (19.1)	39 528 (23.7)	< 0.001
Within recommended gwg	27 (23.5)	1602 (26.1)		205 (24.6)	45 460 (27.3)	
Above recommended gwg	51 (44.3)	2093 (34.0)		378 (45.4)	64 121 (38.4)	
Gestational age (weeks + days) at delivery			< 0.001			< 0.001
34 + 0–36 + 6	22 (19.1)	212 (3.4)		109 (13.1)	5548 (3.3)	
37 + 0–39 + 6	91 (79.1)	5567 (90.6)		529 (63.6)	64 808 (38.8)	
40 + 0–40 + 6	2 (1.7)	186 (3.0)		152 (18.3)	51 958 (31.1)	
41 + 0–41 + 6	0 (0.0)	146 (2.4)		36 (4.3)	33 835 (20.3)	
≥ 42	0 (0.0)	36 (0.6)		6 (0.7)	10 669 (6.4)	
Birthweight (grams)			< 0.001			< 0.001
< 2500	2 (1.7)	199 (3.2)		25 (3.0)	3815 (2.3)	
2500–3999	58 (50.4)	5223 (85.0)		575 (69.1)	141 103 (84.6)	
≥ 4000	55 (47.8)	725 (11.8)		232 (27.9)	21 900 (13.1)	
SGA	2 (1.7)	178 (2.9)	0.462	11 (1.3)	6078 (3.6)	< 0.001
LGA	57 (49.6)	456 (7.4)	< 0.001	218 (26.2)	3331 (2.0)	< 0.001

*Note:* Figures are numbers and percentages if not otherwise specified.

Abbreviations: BMI, Body Mass Index; CS, Caesarean Section; GWG, Gestational Weight Gain; LGA, Large for Gestational Age; SGA, mall for Gestational Age.

^a^ *p*‐value for heterogeneity obtained by Chi‐square analyses.

^b^ *p*‐value obtained by Mann–Whitney *U*‐test.

^c^ According to the American Institute of Medicine's recommendations on gestational weight gain during pregnancy.

Women with pregestational diabetes had more preeclampsia compared to women with no diabetes; both women delivered by elective CS (13.0% vs. 4.3%) and women in trial of labour (2.9% vs. 0.5%). 34.4% of women with pregestational diabetes going into trial of labour were induced compared to 9.9% among women with no diabetes. Among women with pregestational diabetes, 18.6% were delivered by vacuum extraction or forceps compared to 11.2% among women with no diabetes. A more extensive table of the maternal, labour, and neonatal characteristics is presented in Table S4.

Compared to women without diabetes, women with pregestational diabetes were more likely to be delivered with both elective and emergency CS (Table 2).

**TABLE 2:** Indications and rates of elective and emergency caesarean section in women with pregestational diabetes and women with no diabetes (only cephalic presentation, gestational diabetes mellitus excluded).

	Pregestational diabetes *N* = 1546	No diabetes *N* = 237 056	Risk ratio (95% CI)	*p*
	*n* (%)	*n* (%)		
Total caesarean section	535 (34.6)	22 451 (9.5)	3.65 (3.41–3.92)	< 0.001
Elective caesarean section	115 (7.4)	6147 (2.6)	2.87 (2.40–3.43)	< 0.001
Emergency caesarean section (Elective CS excluded)	420/1431 (29.4)	16 304/230909 (7.1)	4.16 (3.83–4.51)	< 0.001
**Indications for elective caesarean section**	***N* = 115**	***N* = 6147**	**RR for certain indication among all elective CS (95% CI)**	
Suspected macrosomia	58 (50.4)	535 (8.7)	5.80 (4.80–7.07)	< 0.001
Preeclampsia	15 (13.0)	266 (4.3)	3.01 (1.85–4.90)	< 0.001
Small for gestational age	2 (1.7)	186 (3.0)	0.58 (0.14–2.29)	0.432
Placenta previa	1 (0.9)	334 (5.4)	0.16 (0.23–1.13)	0.066
Maternal request (O828)	24 (20.9)	3571 (58.1)	0.36 (0.25–0.51)	< 0.001
Other indications than above^a^	15 (13.0)	1255 (20.4)	0.64 (0.40–1.03)	0.064
**Indications for emergency caesarean section**	***N* = 420**	***N* = 16 304**	**RR for certain indication among all emergency CS (95% CI)**	
Fetal distress	134 (31.9)	5858 (35.9)	0.89 (0.77–1.02)	0.099
Obstructive labour/Labour dystocia	91 (21.7)	5620 (34.5)	0.63 (0.52–0.76)	< 0.001
Preeclampsia	103 (24.5)	1341 (8.2)	3.00 (2.50–3.55)	< 0.001
Chorioamnionitis	1 (0.2)	88 (0.5)	0.44 (0.62–3.16)	0.415
Failed induction	8 (1.9)	229 (1.4)	1.36 (0.68–2.73)	0.393
Other indications than above^b^	83 (19.8)	3168 (19.4)	0.93 (0.85–1.02)	0.148

^a^ For example; previous surgery to the uterine wall, pelvic reservoir, non‐reassuring fetal heart rate, unstable fetal position, other intercurrent illness as indication for caesarean section. (elective caesarean section).

^b^ For example; maternal request, placental abruption, umbilical cord prolapse, insufficient pain relief (emergency caesarean section).

Table 3 shows results from Kaplan–Meier analyses, considering right‐censoring because of emergency CS. Displayed are the median (with 95% CI) of labour duration by presence of diabetes and type of start of labour (spontaneous or induced). The results revealed that women with pregestational diabetes had significantly (*p* < 0.001) longer duration of labour, irrespective of type of labour start. The differences between diabetic and non‐diabetic women were 0.62, 1.72, and 0.85 h among all, induced, and spontaneously started labours, respectively (Table 3 and Figure S3a,b).

**TABLE 3:** Duration of labour by presence of pregestational diabetes in spontaneous and induced labour. Results from Kaplan–Meier analyses, considering censoring due to emergency caesarean section.

	*N*	Duration of labour (hours)		Comparison medians pregestational diabetes vs. no diabetes		
		Median	95% CI	Difference	95% CI	*p*
All						
Pregestational diabetes	832	9.22	8.71–9.72	0.62	0.11–1.13	< 0.001
No diabetes	166 818	8.60	8.57–8.63			
Induction of labour						
Pregestational diabetes	286	8.92	8.15–9.68	1.72	0.94–2.49	< 0.001
No diabetes	16 514	7.20	7.11–7.29			
Spontaneous start of labour						
Pregestational diabetes	546	9.60	8.96–10.24	0.85	0.20–1.50	< 0.001
No diabetes	150 304	8.75	8.72–8.78			

**FIGURE 1:**
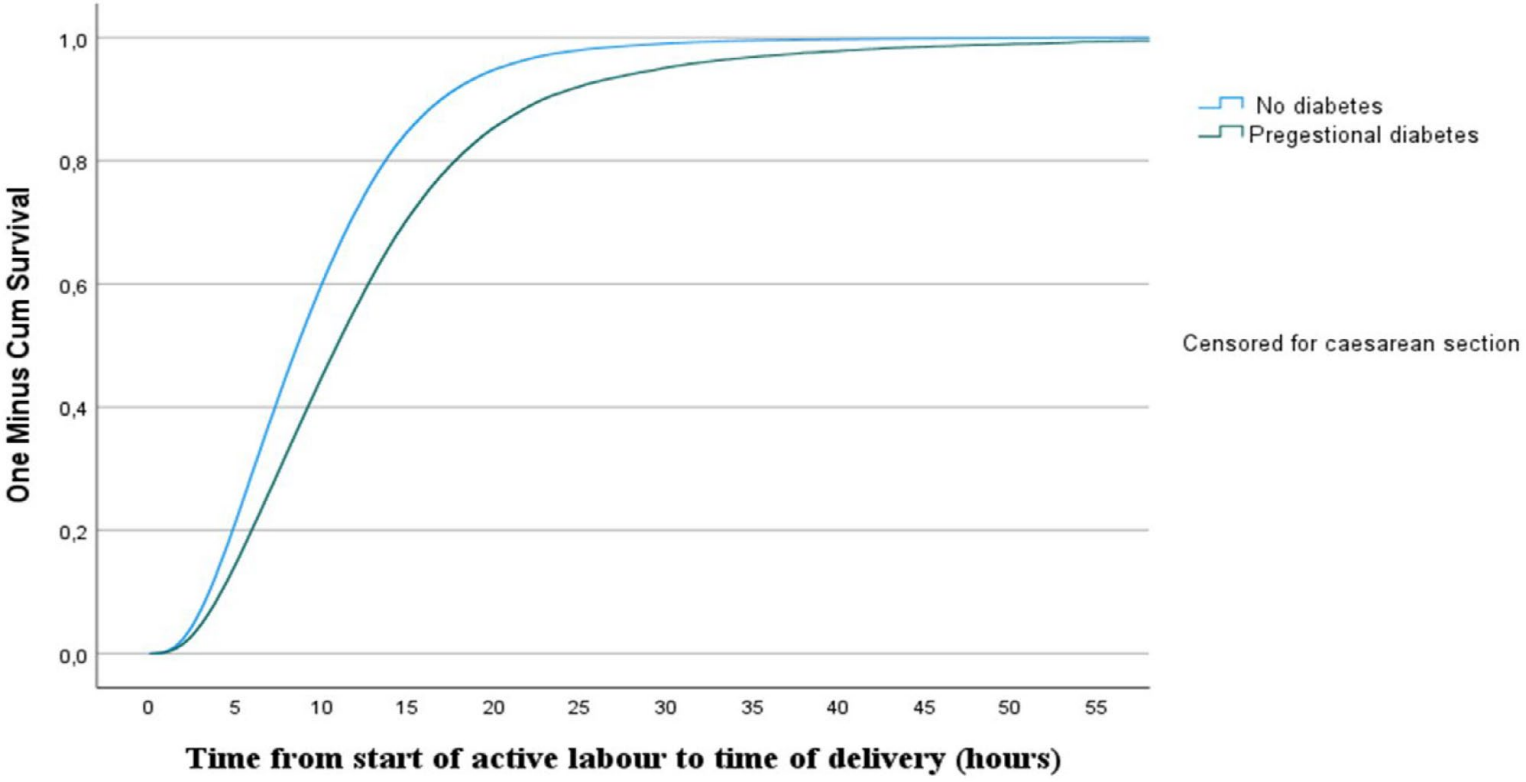
Cox regression analysis of duration of active labour in women with pregestational diabetes compared to women with no type of diabetes. Adjustments were made for maternal body mass index, maternal age, smoking in early pregnancy, gestational week at delivery and induction of labour.

The Cox regression analyses, adjusted for confounders and taking censoring due to emergency CS into account, showed that women with pregestational diabetes had a significantly longer DAL (Figure 1). Women with pregestational diabetes also had a decreased chance of a vaginal delivery at a certain time‐point compared to women without diabetes, with an aHR of 0.65 (95% CI 0.60–0.70), *p* < 0.001. Adding the mediator infant birth weight to the Cox regression analyses changed the estimate marginally (aHR 0.72; 95% CI 0.69–0.78; *p* < 0.001) (Table 4). The estimates for spontaneously started labours and induced were almost identical.

**TABLE 4:** Adjusted Hazard Ratio for birth, pregestational diabetes versus no diabetes. Results from Cox regression analyses.

	Adjusted hazard ratio for birth, Pregestational diabetes vs. no diabetes			
	Adjusted for maternal characteristics^a^		Also adjusted for infant birth weight	
	aHR	95% CI	aHR	95% CI
All	0.65	0.60–0.70	0.72	0.67–0.78
Induction of labour	0.62	0.55–0.71	0.71	0.63–0.81
Spontaneous onset of labour	0.68	0.62–0.75	0.75	0.68–0.82

^a^ Adjusted for maternal age, maternal body mass index, smoking in early pregnancy, and gestational week at delivery. The group “all” was also adjusted for induction of labour.

The indications and rates for elective and emergency CS for women with pregestational diabetes and those without diabetes are shown in Table 2. The elective CS rate was 7.4% in women with pregestational diabetes and 2.6% in women with no diabetes, with a risk ratio (RR) of 2.87 (95% CI 2.40–3.43). Corresponding rates for emergency CS were 27.2% versus 6.9% with a RR of 4.16 (95% CI 3.83–4.51).

## 4 Discussion

### 4.1 Main Findings

In this nation‐wide population‐based cohort of nulliparous women, we observed a longer duration of both spontaneous and induced active labour in women with pregestational diabetes, as well as a reduced likelihood of vaginal delivery at any given time during active labour, compared with women without diabetes.

### 4.2 Strengths and Limitations

The strengths of this study include the large dataset of nulliparous women. Furthermore, the study is nationwide, which adds diversity and increases the generalizability of the findings. Also, register‐based research is ethically safe.

In contrast to previous studies [12, 13], we exclusively included women with pregestational diabetes, given that this group represents a higher risk profile compared to those with gestational diabetes, both during pregnancy and labour. We chose to exclude women with GDM since the longer duration of labour in women with GDM might be contributed to by the same mechanisms as in women with pregestational diabetes [14]. By excluding women with GDM, the comparison between women with pregestational diabetes and those with no diabetes appeared more accurate.

Furthermore, we included women giving birth preterm (≥ 34^+0^ gestational weeks) since preterm elective CS or IOL is common in women with pregestational diabetes due to pregnancy complications. Including these cases enhances the clinical relevance of the results and increases the size of the study population. Another strength is the methodology using Cox regression models, which enables adjustments for relevant confounding factors and censoring for emergency CS instead of including only vaginal deliveries.

The detailed data on baseline evaluation of maternal comorbidity, socioeconomic factors and continuous registration of pregnancy complications enabled adjustments for possible confounding factors, such as maternal BMI in early pregnancy [19]. However, we cannot exclude the possibility that the adjusted estimates could have been influenced by covariates not accounted for in the current analysis, like glycaemic control and HbA1c levels.

General limitations of large register studies include the risk of errors in recorded data and missing values. However, drop‐out analyses and characteristics of women with missing data on start of active labour did not show any clinically relevant demographic difference.

### 4.3 Interpretation

There are a few existing studies on women with pregestational diabetes and duration of labour, with varying outcomes. Since these studies [12, 13] include both women with GDM and women with pregestational diabetes, the results are challenging to interpret. In addition, neither of these studies includes women with emergency CS, which may lead to selection bias.

A study including 122 women with GDM or pregestational diabetes excluding those with emergency CS [12], found that women with diabetes and IOL had longer time to delivery and lower delivery rates within 36 or 48 h due to a prolonged latent phase. Unlike our findings, the duration of active labour was similar to that in women without diabetes. This discrepancy may partly reflect the exclusion of women with diabetes who underwent emergency CS and potentially had longer labour duration. Another study by Timofeev et al. demonstrated that active labour progression and duration were similar in 458 nulliparous and 1079 multiparous women with pregestational diabetes or GDM compared to normal controls, matched by neonatal birth weight, parity and maternal BMI [13]. However, only vaginal deliveries with normal neonatal outcomes were included.

In a previous national cohort study, we demonstrated that women with GDM experienced longer DAL compared to women without GDM [14]. In the current study, the difference in DAL between women with pregestational diabetes and those without diabetes was even more pronounced. This disparity may reflect the duration of illness, including the greater glycaemic variability often observed in women with pregestational diabetes, which could potentially contribute to these findings.

Among women with pregestational diabetes, presumably only those with well‐regulated glycaemic control and a low risk for macrosomia are selected to go into a TOL. Even so, women with pregestational diabetes have a longer DAL in the present study. Encouragingly for labour ward staff, most TOL in pregestational diabetes achieved a vaginal delivery with only 29% requiring an emergency CS. This indicates that TOL is worthwhile in this group.

We also noted that among the women with pregestational diabetes undergoing TOL, 26.2% of the neonates were born LGA and 27.9% of the neonates weighed more than 4000 g. The management of women with pregestational diabetes with suspected fetal macrosomia and/or LGA represents a significant challenge in contemporary obstetrics, especially regarding the timing and mode of delivery.

Our study indicates that pregestational diabetes during pregnancy may independently serve as a risk factor for prolonged DAL. There are theories that hyperglycaemia may affect the myometrial contractility on a cellular level. Al Qathani et al. studied myometrial contractility on lower uterine segment biopsies from women with pregestational diabetes, GDM, or without diabetes undergoing elective CS. They found that women with diabetes had less uterine muscle mass, reduced calcium channel expression, reduced intracellular calcium, and a decreased contraction amplitude and duration in vitro, which may translate into inferior uterine contractility. The authors concluded that these factors significantly contribute to the increased emergency CS rate in women with diabetes [10].

The elective and emergency CS rates were 3–4 times higher among women with pregestational diabetes than for women without diabetes in our study. However, both elective and emergency CS rates in this group were lower in our study compared to previous studies [4, 7, 8, 9, 20]. Our findings may reflect the generally low CS rates in Sweden from a global perspective [21].

Suspected fetal macrosomia was the most common indication for elective CS among women with pregestational diabetes in our study, which is consistent with previous studies [9]. The definition of fetal macrosomia varies between 4000 and 4500 g internationally [22], and differs from the term LGA which reflects fetal growth in relation to gestational week and sex [17]. In a study by Fisher et al., women with type 1 diabetes (T1D) scheduled for elective CS were characterised by poorer glycaemic control and higher estimated fetal size than those offered a TOL [20].

Previous studies analysing indications for emergency CS in women with pregestational diabetes have shown various results. A Finnish study with a similar population found that fetal distress was the most common CS indication in T1D, accounting for 50% of cases [4], consistent with our findings. In contrast, another study reported failed IOL as the leading indication in 50% of women with T1D [9], compared to only 2% in the present study. Variations in definitions of CS indications and obstetric practices across countries may explain these discrepancies.

## 5 Conclusion

The prolonged labour duration in women with pregestational diabetes highlights the significance of the labour ward staff's support and patience in managing diabetic parturients, potentially allowing more time before diagnosing labour dystocia in this population. An extended period of active labour may influence how women perceive their birth experience and emphasises the importance of providing these women with comprehensive information prior to labour.

The primary reason for elective CS being suspected macrosomia in women with pregestational diabetes underscores the complications associated with this condition. These findings could be of interest to all health care professionals involved in optimising the care of women with pregestational diabetes before and during pregnancy and in labour.

The prolonged duration of active labour observed in women with pregestational diabetes warrants further investigation, particularly in light of new evidence and guidelines regarding the onset of active labour and labour progression. Furthermore, a deeper understanding of how pregestational diabetes affects myometrial contractility and labour duration is essential to improve obstetric management of these women.

## Author Contributions

S.N., M.B. and K.K. designed and planned the study. K.K. and S.N. performed the statistical analyses. S.N., M.B., K.K., S.C., and C.L. contributed to the analysis and interpretation of data for the work. S.N. wrote the manuscript. M.B., K.K., C.L. and S.C. critically revised the manuscript. All authors gave final approval and agreed to be accountable for all aspects of the work, ensuring integrity and accuracy. M.B. is the guarantor.

## Ethics Statement

This study was approved by the Regional Ethical Review Board in Linköping, Sweden (2018/464‐31), date of approval 2018‐11‐06.

## Conflicts of Interest

The authors declare no conflicts of interest.

## Supporting information


Figure S1.

Figure S2.

Figure S3.

Table S1.

Table S2.

Table S3.

Table S4.

## Data Availability

Data in this study cannot be made generally available. The data included in this study were based on Swedish electronic medical records and national health care registers. According to Swedish law, data‐sharing is not allowed, as individual‐level data in the medical records and registers can only be accessed through secure servers, and only the export of aggregated data is permitted.
