# Supplementary material for: Pregestational Diabetes and Duration of Active Labour Compared With Non‐Diabetic Women: A Population‐Based Cohort Study
Source: BJOG. 2025 Jul 7;132(11):1635–43. doi: 10.1111/1471-0528.18276 (PMC12411654; doi:10.1111/1471-0528.18276)
Supplement: Supplementary file 1 — Figure S1. [file BJO-132-1635-s001.docx]

Figure S1: Flowchart of the study population

Pregestational diabetes n=420

Included in the subanalysis of indications of emergency caesarean section:

n= 16724

No diabetes n=16304

No diabetes n=166818

Pregestational diabetes

n=832

Pregestational diabetes n=599

Final study population for analyses of duration of active labour

n=167650

Pregestational diabetes n=115

| Excluded due to missing value or faulty data on start of active labour  n= 64690 |
| --- |
|  |

No diabetes n=64091

No diabetes n=6147

Included in the analyses of indications of elective caesarean section

| Excluded from analysis of duration of active labour: |
| --- |
| Elective caesarean section n = 6262 |
|  |

All nulliparae women, singleton pregnancy, ≥34+0 gestational weeks and delivered between Jan 1 2014 and May 30 2020 n=243537

| Excluded: |
| --- |
| Gestational diabetes mellitus n = 4935 |
|  |

Data restricted to nulliparae with trial of labour

n=232340

Nulliparae women, (gestational diabetes mellitus excluded)

n= 238602
