# Supplementary material for: Pregestational Diabetes and Duration of Active Labour Compared With Non‐Diabetic Women: A Population‐Based Cohort Study
Source: BJOG. 2025 Jul 7;132(11):1635–43. doi: 10.1111/1471-0528.18276 (PMC12411654; doi:10.1111/1471-0528.18276)
Supplement: Supplementary file 2 — Figure S2. [file BJO-132-1635-s004.pptx]

## Slide 1
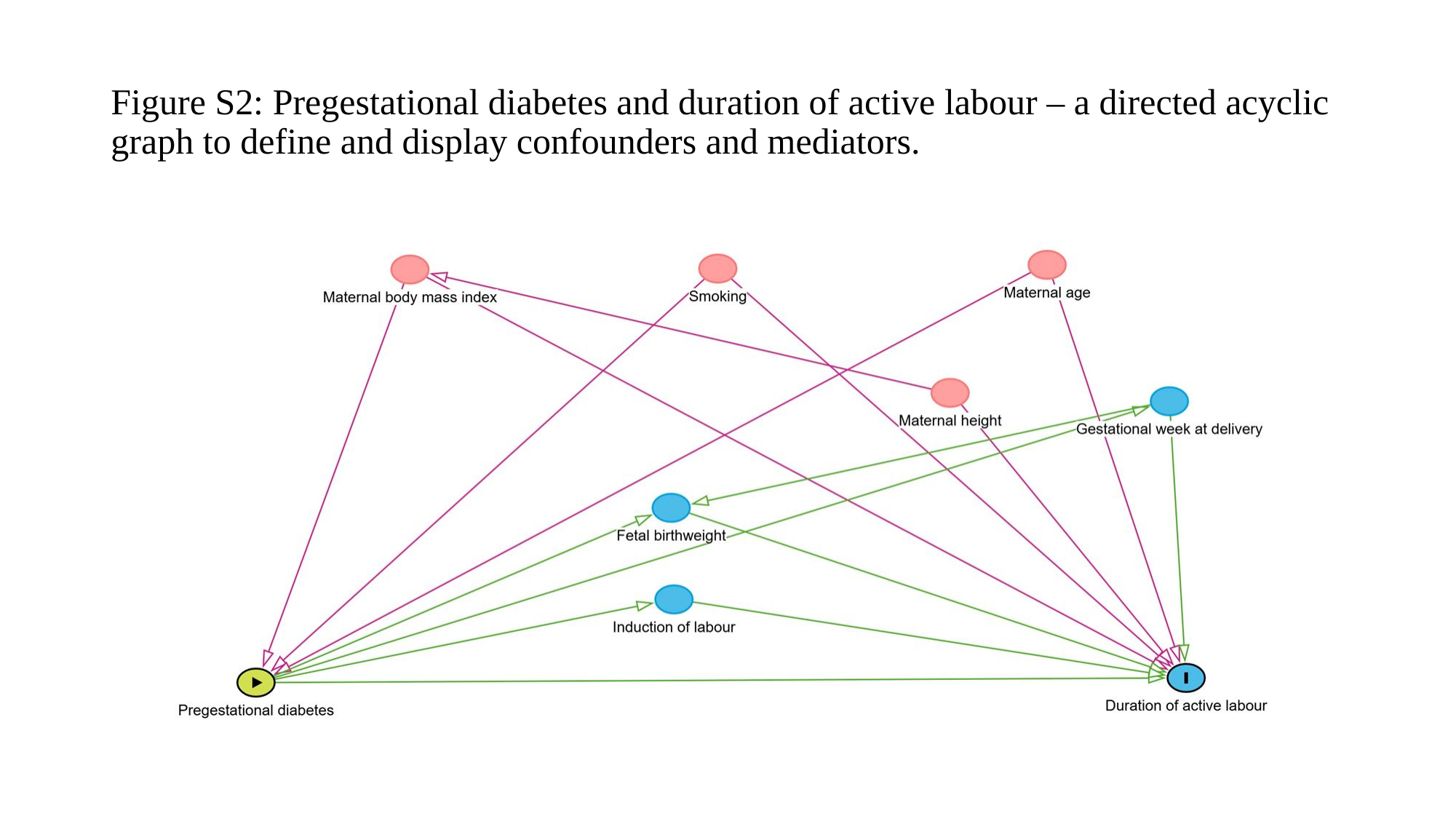

# Figure S2: Pregestational diabetes and duration of active labour – a directed acyclic graph to define and display confounders and mediators.
