# Supplementary material for: Pregestational Diabetes and Duration of Active Labour Compared With Non‐Diabetic Women: A Population‐Based Cohort Study
Source: BJOG. 2025 Jul 7;132(11):1635–43. doi: 10.1111/1471-0528.18276 (PMC12411654; doi:10.1111/1471-0528.18276)
Supplement: Supplementary file 3 — Figure S3. [file BJO-132-1635-s003.zip › Figure S3b.pptx]

## Slide 1
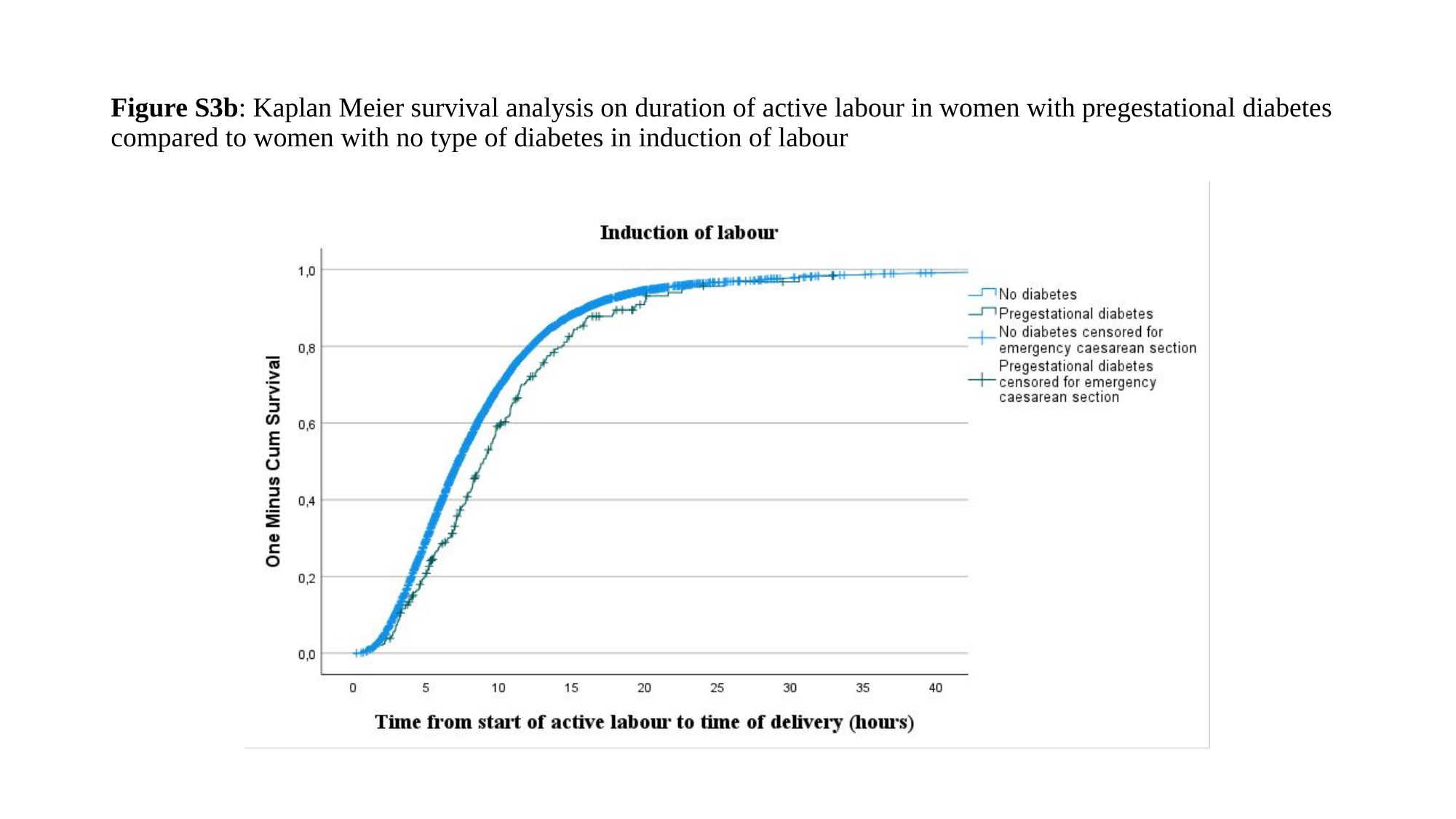

# Figure S3b: Kaplan Meier survival analysis on duration of active labour in women with pregestational diabetes compared to women with no type of diabetes in induction of labour
