# Supplementary material for: Pregestational Diabetes and Duration of Active Labour Compared With Non‐Diabetic Women: A Population‐Based Cohort Study
Source: BJOG. 2025 Jul 7;132(11):1635–43. doi: 10.1111/1471-0528.18276 (PMC12411654; doi:10.1111/1471-0528.18276)
Supplement: Supplementary file 4 — Table S1. [file BJO-132-1635-s005.docx]

**Table S1:** List of variables from the Swedish Pregnancy Register and the mode of extracting the variables.

|  | Checkbox* | ICD-code | Manually registered | Calculated |
| --- | --- | --- | --- | --- |
| Pregestational diabetes | x | O24.0B, O24.0C, O24.0D, O24.0F, O24.0X, O24.1, E10.-, E11.- |  |  |
| Age at delivery |  |  |  | x |
| Country of birth | x |  |  |  |
| Education level | x |  |  |  |
| Body mass index (BMI) in early pregnancy | x |  |  |  |
| Maternal height | x |  |  |  |
| Gestational weight gain |  |  |  | x |
| Smoking in early pregnancy | x |  |  |  |
| Support for fear of childbirth during pregnancy | x |  |  |  |
| Treatment for psychiatric disorder during pregnancy | x |  |  |  |
| Hypertension | x | O10.- |  |  |
| Preeclampsia |  | O14.- |  |  |
| Gestational age at delivery. | x |  |  |  |
| Labour onset type (spontaneous or induction) | x |  |  |  |
| The time of start of contractions |  |  | x |  |
| The time of start of the active phase of labour |  |  | x |  |
| The time of delivery |  |  | x |  |
| Usage of epidural anesthesia | x |  |  |  |
| Usage of oxytocin | x | DT037 |  |  |
| Fetal presentation (crown, occiput posterior or other) | x |  |  |  |
| Instrumental delivery (vacuum extraction/forceps) | x |  |  |  |
| Mode of delivery (non-instrumental vaginal delivery, instrumental delivery, CS) | x |  |  |  |
| Indication for elective caesarean section |  | Fetal macrosomia (O33.9 + O36.6), Preeclampsia (O14.-), Intrauterine growth restriction (O36.5), placenta previa (O44.-), O82.8 |  |  |
| Indication for emergency caesarean section |  | Fetal distress (O68.9), Labour dystocia (O62.0, O62.1, O62.9, O66.9), Preeclampsia (O14.-), chorioamnionitis (O41.1), Failed induction (O61.0B, O61.1B) |  |  |
| Fetal birthweight |  |  | x |  |
| Small for gestational age (SGA) |  |  |  | x |
| Large for gestational age (LGA) |  |  |  | x |

*An automatically transferred filled checkbox from the medical record to the pregnancy registry.
