# Supplementary material for: Pregestational Diabetes and Duration of Active Labour Compared With Non‐Diabetic Women: A Population‐Based Cohort Study
Source: BJOG. 2025 Jul 7;132(11):1635–43. doi: 10.1111/1471-0528.18276 (PMC12411654; doi:10.1111/1471-0528.18276)
Supplement: Supplementary file 5 — Table S2. [file BJO-132-1635-s006.docx]

Table S2: Order in the hierarchy for elective and emergency caesarean sections

| Elective caesarean section |  | Emergency caesarean section |
| --- | --- | --- |
| 1.Suspected macrosomia |  | 1.Fetal distress |
| 2. Preeclampsia |  | 2. Obstructive labour/Labour dystocia |
| 3. Small for gestational age |  | 3. Preeclampsia |
| 4. Placenta previa |  | 4. Chorioamnionitis |
| 5. Maternal request |  | 5. Failed induction |
| 6. Other indications than aboveª |  | 6. Other indications than aboveᵇ |

ª For example; Previous surgery to the uterine wall, pelvic reservoir, non reassuring fetal heart rate, unstable fetal position, other intercurrent illness as indication for CS

ᵇFor example; maternal request, placental abruption, umbilical cord prolapse, insufficient pain relief
