# Supplementary material for: Pregestational Diabetes and Duration of Active Labour Compared With Non‐Diabetic Women: A Population‐Based Cohort Study
Source: BJOG. 2025 Jul 7;132(11):1635–43. doi: 10.1111/1471-0528.18276 (PMC12411654; doi:10.1111/1471-0528.18276)
Supplement: Supplementary file 6 — Table S3. [file BJO-132-1635-s007.docx]

Table S3: Drop-out analyses by presence of pregestational diabetes and maternal characteristics in trial of labour deliveries. Included in the study population were deliveries with available information on start of active labour. Not included were women with missing information on start of active labour.

|  |  | Included  N=167 650 | | Not included  N=64 690 | | p-value^A^ |
| --- | --- | --- | --- | --- | --- | --- |
|  |  | n | (%) | n | (%) |  |
| Diabetes | |  |  |  |  | <0.001 |
|  | Yes | 832 | ( 0.5) | 599 | ( 0.9) |  |
|  | No | 166818 | (99.5) | 64091 | (99.1) |  |
| Age (years) | |  |  |  |  | <0.001 |
|  | <20 | 3590 | ( 2.1) | 1575 | ( 2.4) |  |
|  | 20-35 | 146552 | (87.4) | 54535 | (84.3) |  |
|  | 35-39 | 14831 | ( 8.8) | 6808 | (10.5) |  |
|  | 40+ | 2638 | ( 1.6) | 1749 | ( 2.7) |  |
|  | Age not known | 39 | ( 0.0) | 23 | ( 0.0) |  |
| Country of birth | |  |  |  |  | <0.001 |
|  | Nordic countries | 119414 | (71.2) | 43710 | (67.6) |  |
|  | Europe or western world | 10836 | ( 6.5) | 4238 | ( 6.6) |  |
|  | Outside EU/western world | 37400 | (22.3) | 16742 | (25.9) |  |
| Educational level | |  |  |  |  | <0.001 |
|  | No schooling | 1237 | ( 0.7) | 657 | ( 1.0) |  |
|  | ≤9 years | 7061 | ( 4.2) | 3079 | ( 4.8) |  |
|  | 10-12 years | 53712 | (32.0) | 20388 | (31.5) |  |
|  | University | 78609 | (46.9) | 29133 | (45.0) |  |
|  | Education level unknown | 27031 | (16.1) | 11433 | (17.7) |  |
| BMI (kg/m²)) | |  |  |  |  | <0.001 |
|  | <18.5 | 4834 | ( 2.9) | 1759 | ( 2.7) |  |
|  | 18.5-24.9 | 95360 | (56.9) | 34247 | (52.9) |  |
|  | 25-29.9 | 34630 | (20.7) | 14315 | (22.1) |  |
|  | 30-34.9 | 10940 | ( 6.5) | 5037 | ( 7.8) |  |
|  | 35+ | 4087 | ( 2.4) | 2070 | ( 3.2) |  |
|  | BMI not known | 17799 | (10.6) | 7262 | (11.2) |  |
| Gestational weight gain | |  |  |  |  | <0.001 |
|  | Below recommendation | 39687 | (23.7) | 14520 | (22.4) |  |
|  | Recommended | 45665 | (27.2) | 16542 | (25.6) |  |
|  | Over recommendation | 64499 | (38.5) | 26366 | (40.8) |  |
|  | Not known | 17799 | (10.6) | 7262 | (11.2) |  |
| Smoking | |  |  |  |  | <0.001 |
|  | Non smoking | 149734 | (89.3) | 57396 | (88.7) |  |
|  | Smoking | 6458 | ( 3.9) | 2704 | ( 4.2) |  |
|  | Not known | 11458 | ( 6.8) | 4590 | ( 7.1) |  |
| Snuff |  |  |  |  |  | 0.072 |
|  | No snuff use | 165976 | (99.0) | 63985 | (98.9) |  |
|  | Snuff use | 1552 | ( 0.9) | 663 | ( 1.0) |  |
|  | Not known | 122 | ( 0.1) | 42 | ( 0.1) |  |
| Mental health | |  |  |  |  |  |
|  | Fear of labour | 6712 | ( 4.0) | 3292 | ( 5.1) | <0.001 |
|  | Any psychological diagnosis | 9878 | ( 5.9) | 4295 | ( 6.6) | <0.001 |

^A^ Obtained from Chi-2 analyses calculating the overall heterogeneity within each domain.
