# Supplementary material for: Pregestational Diabetes and Duration of Active Labour Compared With Non‐Diabetic Women: A Population‐Based Cohort Study
Source: BJOG. 2025 Jul 7;132(11):1635–43. doi: 10.1111/1471-0528.18276 (PMC12411654; doi:10.1111/1471-0528.18276)
Supplement: Supplementary file 7 — Table S4. [file BJO-132-1635-s002.docx]

**Table S4: Maternal, labour and neonatal characteristics for women with trial of labour (with available data on start of active labour) and women undergoing elective caesarean section**

|  |  | **Elective caesarean section** | | | **Trial of labour** | | |
| --- | --- | --- | --- | --- | --- | --- | --- |
|  |  | **Pre-gestational diabetes**  N= 115 | **No diabetes**  N= 6147 | **p-value^a^** | **Pre-gestational**  **diabetes**  N= 832 | **No diabetes**  N= 166818 | **p-value^a^** |
|  |  | n (%) | n (%) |  | n (%) | n (%) |  |
| **Age** (years) | |  |  | 0.560 |  |  | 0.088 |
|  | <20 | 0 (0.0) | 52 (0.8) |  | 10 (1.2) | 3580 (2.1) |  |
|  | 20-34 | 80 (69.6) | 3946 (64.2) |  | 718 (86.3) | 145834 (87.4) |  |
|  | 35-39 | 27 (23.5) | 1484 (24.1) |  | 87 (10.5) | 14744 (8.8) |  |
|  | ≥40 | 1 (0.9) | 122 (2.0) |  | 0 ( 0.0) | 102 (0.1) |  |
|  | Age unknown | 0 (0.0) | 0 (0.0) |  | 0 ( 0.0) | 39 (0.0) |  |
| **Country of birth** | |  |  | 0.015 |  |  | 0.015 |
|  | Nordic countries | 86 (74.8) | 4231 (68.8) |  | 593 (71.3) | 118821 (71.2) |  |
|  | Other EU and USA | 3 (2.6) | 388 (6.3) |  | 35 (4.2) | 10801(6.5) |  |
|  | Remaining/other countries | 26 (22.6) | 1528 (24.9) |  | 204 (24.5) | 37196 (22.3) |  |
| **Education level** | |  |  | 0.026 |  |  | 0.425 |
|  | no schooling | 1 (0.9) | 28 (0.5) |  | 4 (0.5) | 1233 (0.7) |  |
|  | ≤9 years | 1 (0.9) | 191 (3.1) |  | 38 (4.6) | 7023 (4.2) |  |
|  | 10-12 years | 38 (33.0) | 1575 (25.6) |  | 256 (30.8) | 53456 (32.0) |  |
|  | University | 44 (38.3) | 3268 (53.2) |  | 342 (41.1) | 78267 (46.9) |  |
|  | Education level unknown | 31 (27.0) | 1085 (17.7) |  | 192 (23.1) | 26839 (16.1) |  |
| **BMI (kg/m²) in early pregnancy** | |  |  | <0.001 |  |  | <0.001 |
|  | <18.5 | 1 (0.9) | 197 (3.2) |  | 9 (1.1) | 4825 (2.9) |  |
|  | 18.5-24.9 | 38 (33.0) | 3215 (52.3) |  | 355 (42.7) | 95005 (57.0) |  |
|  | 25-29.9 | 33 (28.7) | 1217(19.8) |  | 229 (27.5) | 34401 (20.6) |  |
|  | 30-34.9 | 20 (17.4) | 462 (7.5) |  | 100 (12.0) | 10840 (6.5) |  |
|  | ≥35 | 7 (6.1) | 164 (2.7) |  | 49 (5.9) | 4038 (2.4) |  |
|  | BMI unknown | 16 (13.9) | 892 (14.5) |  | 90 (10.8) | 17709 (10.6) |  |
| **Gestational weight gain (gwg)b** | |  |  |  |  |  |  |
|  | Below recommended gwg | 21 (18.3) | 1560 (25.4) | 0.049 | 159 (19.1) | 39528 (23.7) | <0.001 |
|  | Within recommended gwg | 27 (23.5) | 1602 (26.1) |  | 205 (24.6) | 45460 (27.3) |  |
|  | Above recommended gwg | 51 (44.3) | 2093 (34.0) |  | 378 (45.4) | 64121 (38.4) |  |
|  | Gwg unknown | 16 (13.9) | 892 (14.5) |  | 90 (10.8) | 17709 (10.6) |  |
| **Smoking in early pregnancy** | |  |  | 0.371 |  |  | 0.049 |
|  | No | 96 (83.5) | 5532 (90.0) |  | 734 (88.2) | 149000 (89.3) |  |
|  | Yes | 6 (5.2) | 237 (3.9) |  | 43 (5.2) | 6415 (3.8) |  |
|  | Smoking habits unknown | 13 (11.3) | 378 (6.1) |  | 55 (6.6) | 11403 (6.8) |  |
| **Support for fear of childbirth during pregnancy** | | 25 (21.7) | 2682 (43.6) | <0.001 | 28 (3.4) | 6684 (4.0) | 0.345 |
| **Treated for psychiatric disorder during pregnancy** | | 10 (8.7) | 759 (12.3) | 0.237 | 53 (6.4) | 9825 (5.9) | 0.560 |
| **Gestational age (weeks+days) at delivery** | |  |  | <0.001 |  |  | <0.001 |
|  | 34+0 - 36+6 | 22 (19.1) | 212 (3.4) |  | 109 (13.1) | 5548 (3.3) |  |
|  | 37+0 - 39+6 | 91 (79.1) | 5567 (90.6) |  | 529 (63.6) | 64808 (38.8) |  |
|  | 40+0 - 40+6 | 2 (1.7) | 186 (3.0) |  | 152 (18.3) | 51958 (31.1) |  |
|  | 41+0 - 41+6 | 0 (0.0) | 146 (2.4) |  | 36 (4.3) | 33835 (20.3) |  |
|  | ≥42 | 0 (0.0) | 36 (0.6) |  | 6 (0.7) | 10669 (6.4) |  |
| **Preeclampsia** | | 15 (13.0) | 266 (4.3) | <0.001 | 24 (2.9) | 813 (0.5) | <0.001 |
| **Occiput posterior** | |  |  |  | 27 (3.2) | 5143 (3.1) | 0.791 |
| **Induction of labour** | |  |  |  | 286 (34.4) | 16535 (9.9) | <0.001 |
| **Delivery mode** | |  |  |  |  |  | <0.001 |
|  | Emergency CS |  |  |  | 125 (15.0) | 6652 (3.9) |  |
|  | VE/Forceps |  |  |  | 155 (18.6) | 18688 (11.2) |  |
| **Birthweight (grams)** | |  |  | <0.001 |  |  | <0.001 |
|  | < 2500 | 2 (1.7) | 199 (3.2) |  | 25 (3.0) | 3815 (2.3) |  |
|  | 2500-3999 | 58 (50.4) | 5223 (85.0) |  | 575 (69.1) | 141103 (84.6) |  |
|  | ≥ 4000 | 55 (47.8) | 725 (11.8) |  | 232 (27.9) | 21900 (13.1) |  |
| **SGA** | | 2 (1.7) | 178 (2.9) | 0.462 | 11 (1.3) | 6078 (3.6) | <0.001 |
| **LGA** | | 57 (49.6) | 456 (7.4) | <0.001 | 218 (26.2) | 3331 (2.0) | <0.001 |

BMI = Body Mass Index, GWG = Gestational Weight Gain, CS = Caesarean Section, VE = Vacuum Extraction, SGA = Small for Gestational Age, LGA = Large for Gestational Age

^a^ p-value for heterogeneity obtained by Chi-square analyses

^b^ According to the American Institute of Medicine´s recommendations on gestational weight gain during pregnancy.
